# Cardiopulmonary phenotype associated with human *PHD2* mutation

**DOI:** 10.14814/phy2.13224

**Published:** 2017-04-10

**Authors:** Nick P. Talbot, Thomas G. Smith, George M. Balanos, Keith L. Dorrington, Patrick H. Maxwell, Peter A. Robbins

**Affiliations:** ^1^ Department of Physiology, Anatomy & GeneticsUniversity of OxfordOxfordUnited Kingdom; ^2^ School of Sport, Exercise and Rehabilitation ScienceUniversity of BirminghamBirminghamUnited Kingdom; ^3^ Cambridge Institute for Medical ResearchUniversity of CambridgeCambridgeUnited Kingdom

**Keywords:** Hypoxia, hypoxia‐inducible factor, prolyl hydroxylase domain protein, pulmonary circulation, ventilation

## Abstract

Oxygen‐dependent regulation of the erythropoietin gene is mediated by the hypoxia‐inducible factor (HIF) family of transcription factors. When oxygen is plentiful, HIF undergoes hydroxylation by a family of oxygen‐dependent prolyl hydroxylase domain (PHD) proteins, promoting its association with the von Hippel‐Lindau (VHL) ubiquitin E3 ligase and subsequent proteosomal degradation. When oxygen is scarce, the PHD enzymes are inactivated, leading to HIF accumulation and upregulation not only of erythropoietin expression, but also the expression of hundreds of other genes, including those coordinating cardiovascular and ventilatory adaptation to hypoxia. Nevertheless, despite the identification of over 50 mutations in the PHD‐HIF‐VHL pathway in patients with previously unexplained congenital erythrocytosis, there are very few reports of associated cardiopulmonary abnormalities. We now report exaggerated pulmonary vascular and ventilatory responses to acute hypoxia in a 35‐year‐old man with erythrocytosis secondary to heterozygous mutation in *PHD2*, the most abundant of the PHD isoforms. We compare this phenotype with that reported in patients with the archetypal disorder of cellular oxygen sensing, Chuvash polycythemia, and discuss the possible clinical implications of our findings, particularly in the light of the emerging role for small molecule PHD inhibitors in clinical practice.

## Introduction

Oxygen‐dependent gene expression is exemplified by erythropoietin (EPO) production, which is mediated by the hypoxia‐inducible factor (HIF) family of transcription factors (Wang and Semenza 1993; Wang et al. 1995). The HIF transcriptional complex is a heterodimer comprising *α* and *β* subunits, the former of which is regulated by oxygen availability mainly through the action of a family of 2‐oxoglutarate‐dependent prolyl hydroxylase domain (PHD) proteins (Epstein et al. 2001). When oxygen is plentiful, hydroxylation of two specific proline residues in the HIF*α* protein promotes its association with the von Hippel‐Lindau (VHL) ubiquitin E3 ligase, leading to its degradation in the ubiquitin‐proteosomal pathway. When oxygen is scarce, the PHD enzymes are inactivated, leading to HIF*α* accumulation and formation of the transcriptional complex through binding to nuclear HIF*β* (Ivan et al. 2001; Jaakkola et al. 2001). Human HIF*α* exists as three isoforms (HIF1*α*, 2*α* and 3*α*), which regulate overlapping sets of genes (Schodel et al. 2011). Similarly, three PHD isoforms have been described (PHD1, 2 and 3), of which PHD2 is the most abundant (Appelhoff et al. 2004).

Since the elucidation of the PHD‐HIF‐VHL pathway, mutations in its constituent proteins have been identified in previously unexplained cases of congenital erythrocytosis. Chuvash polycythemia, for example, is endemic in the Upper Volga region of Russia, with sporadic cases reported elsewhere, and is characterized by severe erythrocytosis. It is now known to be due to a homozygous loss‐of‐function mutation (*598C>T*) in the *VHL* gene, leading to inappropriate HIF*α* stabilization, activation of HIF‐mediated gene transcription and EPO upregulation (Ang et al. 2002). Importantly, this condition highlights the importance of HIF signaling not only for EPO regulation, but also in the regulation of systemic oxygen delivery more broadly. Patients with Chuvash polycythemia display elevated minute ventilation and pulmonary artery pressures at baseline, and profoundly enhanced pulmonary vascular, ventilatory and cardiac responses to acute hypoxia (Bushuev et al. 2006; Smith et al. 2006, 2008b), a phenotype reminiscent of that seen in lowlanders acclimatized to the hypoxia of high altitude.

To date, over 50 oxygen sensing mutations have been identified in patients with congenital erythrocytosis (Bento et al. 2014; Camps et al. 2016; McMullin 2016). The majority are loss‐of‐function mutations in *VHL* or *EGLN1* (*PHD2*), but gain‐of‐function mutations in the *EPAS1* (*HIF2α*) gene have also been described. Cardiopulmonary abnormalities have been reported in Chuvash polycythemia, in two infants with distinct *VHL* mutations (Bond et al. 2011; Sarangi et al. 2014), and in a small number of patients with *HIF2α* mutations (Gale et al. 2008; Formenti et al. 2011). Abnormal cardiopulmonary physiology associated with PHD2 deficiency has also been demonstrated in mice (Bishop et al. 2013; Dai et al. 2016; Hodson et al. 2016; Kapitsinou et al. 2016), but to the best of our knowledge there are no reports of an analogous cardiopulmonary phenotype in PHD‐deficient humans. We now report exaggerated ventilatory and pulmonary vascular responses to hypoxia in a patient with heterozygous *PHD2* mutation.

## Case Report

We assessed the cardiopulmonary phenotype of a 35‐year‐old man who had been diagnosed with polycythemia 6 years earlier, with peak hemoglobin and hematocrit of 191 g/L and 0.54, respectively. As reported previously (Percy et al. 2006), serum EPO was normal despite the elevated hemoglobin, suggesting dysregulation of erythropoietin secretion. Following extensive investigation, this was attributed to heterozygosity for a *950C>G* mutation in the *PHD2* gene, corresponding to a proline‐arginine substitution. In vitro, the mutated PHD protein was less effective at binding and hydroxylating HIF1*α* and HIF2*α*, and in cell culture overexpression of mutant PHD2 was less effective at suppressing HIF reporter gene activity, compared with wild type protein (Percy et al. 2006).

**Figure 1:**
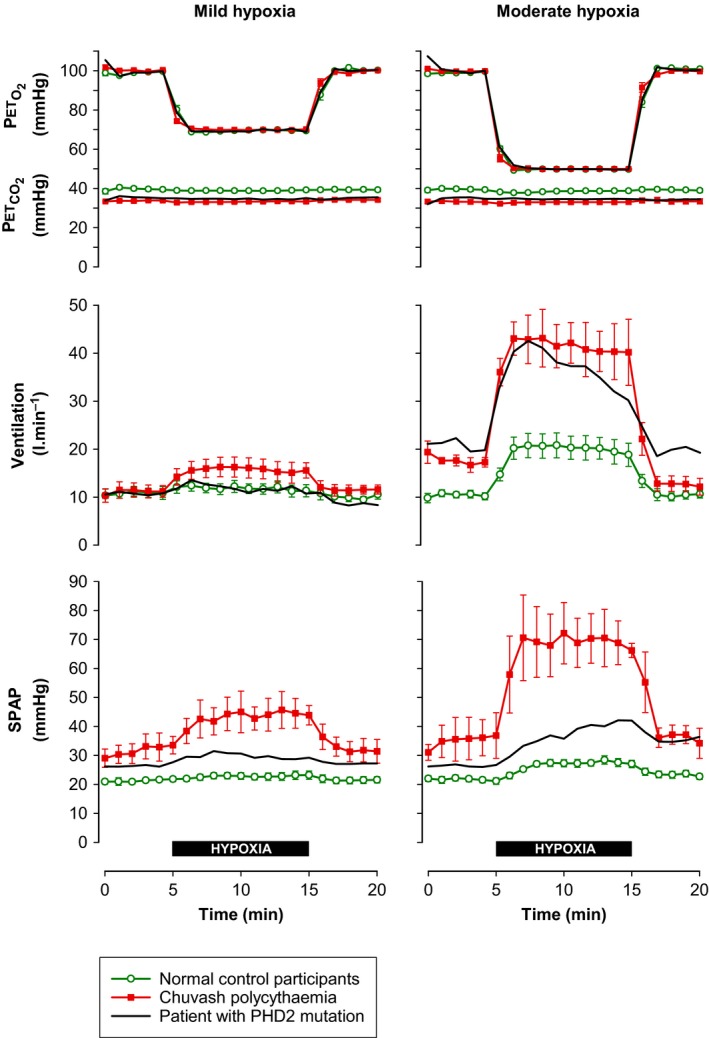
Changes in minute ventilation and systolic pulmonary artery pressure (SPAP) during 10 min of mild and moderate hypoxia in a patient with *PHD2* mutation (current report), compared with previously published (Smith et al. 2006) responses in normal control participants (*n* = 6) and patients with Chuvash polycythemia (*n* = 3). End‐tidal partial pressures of oxygen (PETO_2_) and carbon dioxide (PETCO_2_) were controlled using dynamic end‐tidal forcing. Symbols show mean ± SEM.

The patient was recruited through his consultant physician, and provided written, informed consent for the study, which was approved by the Oxfordshire Clinical Research Ethics Committee. After baseline measurements including spirometry, serum iron indices and arterial blood gas sampling (Table 1), the patient was exposed to a 10‐min period of mild eucapnic hypoxia (end‐tidal PO_2_ 70 mmHg), followed by a 10‐min period of moderate eucapnic hypoxia (end‐tidal PO_2_ 50 mmHg), using a purpose‐built end‐tidal forcing gas control system (Robbins et al. 1982). During hypoxia, minute ventilation was measured continuously, and systolic pulmonary artery pressure (SPAP) and cardiac output were estimated beat‐by‐beat using non‐invasive Doppler echocardiography. This methodology has previously been used to assess a group of three patients with Chuvash polycythemia, plus six healthy control subjects. These results have been reported elsewhere (Smith et al. 2006), but are reproduced in Figure 1 and Table 1, for comparison with the current case.

**Table 1:** Physical characteristics and blood indices in a patient with erythrocytosis secondary to *PHD2* mutation, compared with values from previously published studies on patients with Chuvash polycythemia and healthy control participants (Smith et al. 2006)

	Healthy control participants Mean ± SD (*n* = 6)	Chuvash polycythemia Mean ± SD (*n* = 3)	PHD2‐deficient patient (*n* = 1)
Physical characteristics			
Age (year)	24 ± 5	22 ± 5	35
Height (cm)	173 ± 10	174 ± 5	175
Weight (kg)	73 ± 11	59 ± 10	80
Body mass index (kg/m^2^)	24 ± 2	20 ± 5	26
Full blood count			
Hemoglobin (g/L)	137 ± 17	141 ± 21	178
Hematocrit	0.42 ± 0.05	0.48 ± 0.08	0.52
Mean cell volume (fL)	89 ± 3	64 ± 6^a^	85
Serum iron studies			
Iron (μmol/L)	17 ± 4	4 ± 1^a^	16
Ferritin (ng/ml)	49 ± 37	2 ± 1	193
Transferrin (g/L)	2.6 ± 0.5	3.8 ± 0.3^a^	2.2
Transferrin saturation (%)	30 ± 9	4 ± 1	32
Arterial blood gases			
PO_2_ (mmHg)	99 ± 11	102 ± 3	107
PCO_2_ (mmHg)	40 ± 4	34 ± 2^a^	36
pH (mmHg)	7.38 ± 0.02	7.41 ± 0.02^a^	7.45

a Significant difference compared with control participants (*P* < 0.05, Student's *t*‐test). Mean ± SD.

Spirometry revealed slightly reduced forced expiratory volume in one second (FEV1) and forced vital capacity (FVC) in the PHD2‐deficient patient, compared with age, gender and height adjusted normal values (FEV 74% and FVC 78% predicted, respectively). Similar results were seen previously in patients with Chuvash polycythemia (Smith et al. 2008b). As shown in Table 1, hemoglobin and hematocrit were elevated but mean corpuscular volume and iron studies were normal. Arterial blood gas analysis revealed a PCO_2_ of 36.3 mmHg with borderline alkalosis (pH 7.45), compatible with a mild elevation of basal pulmonary ventilation, relative to metabolism.

Figure 1 shows the ventilatory and pulmonary vascular responses to hypoxia in the PHD2‐deficient patient, compared with previously reported responses in patients with Chuvash polycythemia and in normal volunteers (Smith et al. 2006). With regard to minute ventilation, the patient had a normal response to mild hypoxia, but a substantially enhanced ventilatory response to moderate hypoxia, similar to that seen in the Chuvash patients. With regard to pulmonary hemodynamics, the patient had mildly elevated baseline SPAP, intermediate between the normal and Chuvash groups. During hypoxia, the magnitude of the rise in SPAP in the PHD2‐deficient patient again appeared to be intermediate between the normal response and the exaggerated response seen in Chuvash patients.

## Discussion

From a cardiopulmonary perspective, the best‐characterized oxygen sensing mutations are those associated with Chuvash polycythemia, in whom we have previously described elevated basal ventilation and pulmonary artery pressure, and dramatically enhanced ventilatory, pulmonary vascular and cardiac sensitivity to acute hypoxia (Smith et al. 2006, 2008b). We have also described a small number of patients with *HIF2α* gain‐of‐function mutations, who also have elevated basal ventilation and pulmonary artery pressure, a moderately enhanced pulmonary vascular sensitivity to hypoxia, but a normal ventilatory sensitivity to hypoxia (Formenti et al. 2011). Using similar methodology, we now describe an intermediate phenotype associated with *PHD2* mutation, with mildly elevated basal ventilation and pulmonary artery pressure, a marked increase in the ventilatory sensitivity to hypoxia, but only a relatively modest increase in the pulmonary vascular sensitivity to hypoxia.

We cannot exclude some contribution from the moderately raised blood viscosity to the elevation of pulmonary artery pressure in this case. However, pulmonary hypertension is reported in PHD2‐deficient mice in the absence of polycythemia (Dai et al. 2016; Kapitsinou et al. 2016), and in a previous patient with elevated pulmonary artery pressure and erythrocytosis secondary to *HIF2α* mutation, therapeutic venesection was not associated with any change in pulmonary artery pressure or the magnitude of the pulmonary vascular response to hypoxia (Formenti et al. 2011).

Several factors could contribute to the apparently milder pulmonary vascular phenotype associated with *PHD2* mutation, compared with the Chuvash phenotype. First, PHD2 is reported in cell culture to be of greater importance in the regulation of HIF1*α*, compared with HIF2*α* (Appelhoff et al. 2004), while HIF2*α* appears to be primarily responsible for the pulmonary hypertension associated with Chuvash polycythemia, at least in mice (Hickey et al. 2010). However, despite its apparent preference for HIF1*α* in cell culture, PHD2 appears nonetheless to be an important regulator of HIF2*α* in vivo. Conditional *PHD2* inactivation was recently shown in mice to result in pulmonary hypertension and enhanced ventilatory sensitivity to hypoxia (Hodson et al. 2016; Kapitsinou et al. 2016). The effects of *PHD2* inactivation were dependent upon the presence of HIF2*α*, but were largely unaffected by concomitant loss of HIF1*α*.

Second, it has also been reported in mouse models of Chuvash polycythemia that abnormal erythrocytosis results not only from HIF upregulation, but also from VHL‐dependent, HIF‐independent activation of the JAK‐STAT signaling pathway, leading to enhanced sensitivity of erythroid precursor cells to erythropoietin (Russell et al. 2011). If this pathway also contributes to the pulmonary vascular phenotype in human Chuvash polycythemia, it may not be recapitulated in patients with *PHD2* mutations.

**Figure 2:**
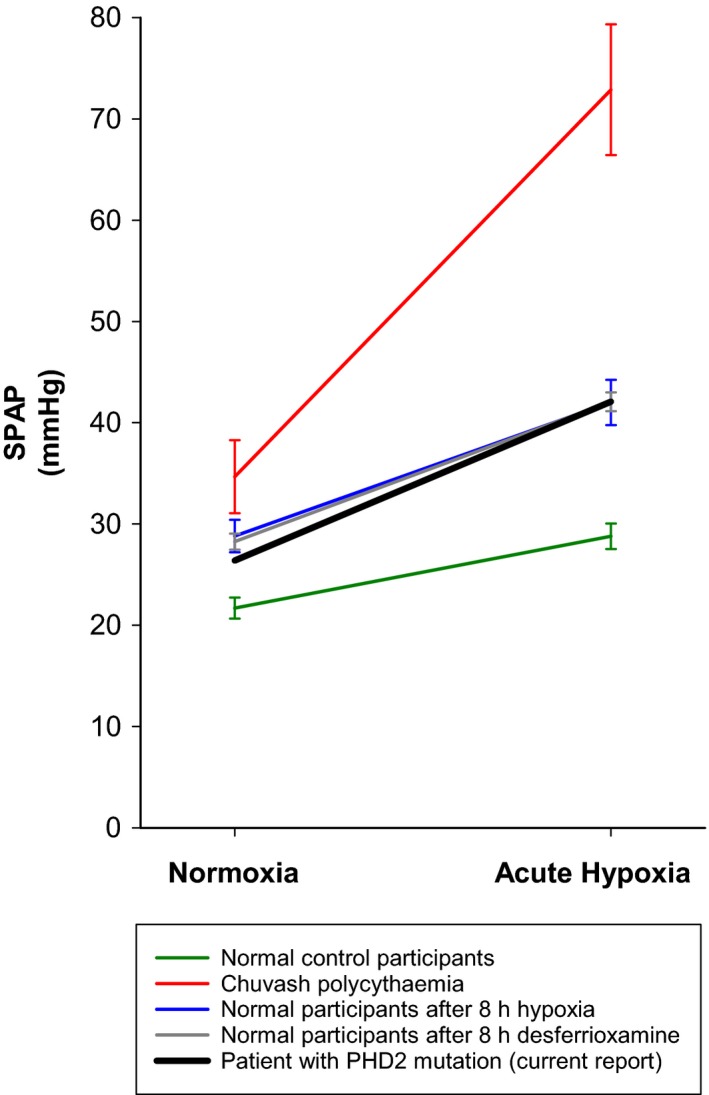
Systolic pulmonary artery pressure (SPAP) breathing room air (Normoxia) and after 10 min of hypoxia (Acute Hypoxia; end tidal PO_2_ 50 mmHg) in a patient with *PHD2* mutation, compared with previously published (Smith et al. 2006, 2008a) responses in normal control participants (*n* = 8), patients with Chuvash polycythemia (*n* = 3), healthy volunteers immediately after prolonged hypoxia (end tidal PO_2_ 55 mmHg for 8 h; *n* = 8), and healthy volunteers immediately after infusion of the iron chelator desferrioxamine mesylate (4 g per 70 kg over 8 h; *n* = 8). Symbols show mean ± SEM.

Third, a notable difference between the previously reported Chuvash patients and the current PHD2‐deficient patient is the presence of severe iron deficiency in the former group. This is likely to reflect the greater severity of erythropoietin dysregulation in patients with Chuvash polycythemia, in whom frequent venesection is required, with consequent iron deficiency. In contrast, the current PHD2‐deficient patient required less frequent venesection, and remained iron replete. Iron deficiency may mimic hypoxia at a cellular level by limiting the intracellular availability of iron, which is required as a co‐factor for the catalytic activity of the PHD proteins. Accordingly, it has recently been reported that iron deficiency in otherwise healthy individuals is associated with enhanced pulmonary vascular responses to hypoxia (Frise et al. 2016) and it has been suggested before that iron deficiency may contribute to the Chuvash pulmonary vascular phenotype (Sable et al. 2012). In this context, Figure 2 shows the striking similarity between the pulmonary vascular response to hypoxia in the current PHD2‐deficient patient and the corresponding response in healthy individuals exposed either to 8 h of hypoxia, or to an 8‐h infusion of the iron chelator desferrioxamine (Smith et al. 2008a).

Finally, our results may have important clinical implications. In combination with previous reports of pulmonary hypertension in patients with *VHL* and *HIF2* mutations (Bushuev et al. 2006; Smith et al. 2006; Gale et al. 2008; Bond et al. 2011; Formenti et al. 2011; Sarangi et al. 2014), our findings support the idea that patients presenting with erythrocytosis secondary to oxygen sensing mutations should be monitored for cardiopulmonary abnormalities (Bento et al. 2014; McMullin 2016). This might be particularly important for those in whom venesection is being considered, or in patients likely to be exposed to hypoxia, such as through ascent to high altitude or during air travel. In Chuvash polycythemia, for example, flight‐induced pulmonary hypertension can develop even when baseline pulmonary artery pressures are not elevated (Smith et al. 2013; Turner et al. 2015). Our results are also significant in the light of the emerging role for small molecule PHD inhibitors in clinical practice, for example to upregulate EPO production in the treatment of patients with renal anemia (Brigandi et al. 2016; Provenzano et al. 2016). Early phase clinical trials investigating these inhibitors have now been completed. Our results raise the possibility that these agents may produce significant alterations in cardiorespiratory control that have not yet been widely considered.

## Conflict of Interest

PHM is a founder, shareholder and director of ReOx Ltd.
